# Dietary Modification with Food Order and Divided Carbohydrate Intake Improves Glycemic Excursions in Healthy Young Women

**DOI:** 10.3390/nu17203194

**Published:** 2025-10-10

**Authors:** Yuki Higuchi, Takashi Miyawaki, Shizuo Kajiyama, Kaoru Kitta, Shintaro Kajiyama, Yoshitaka Hashimoto, Michiaki Fukui, Saeko Imai

**Affiliations:** 1 Department of Food and Nutrition, Kyoto Women’s University, 35, Kitahiyoshi-cho, Imakumano, Higashiyama-ku, Kyoto 605-8501, Japan; 24131104@kyoto-wu.ac.jp (Y.H.); miyawakt@kyoto-wu.ac.jp (T.M.); kittak624@gmail.com (K.K.); 2 Kajiyama Clinic, Kyoto Gojyo Clinic Building, 20-1, Higashionmaeda-cho, Nishinanajyo, Shimogyo-ku, Kyoto 600-8898, Japan; kajiyama-clinic@dream.ocn.ne.jp (S.K.); kaji20091025abcd@gmail.com (S.K.); 3 Department of Endocrinology and Metabolism, Graduate School of Medical Science, Kyoto Prefectural University of Medicine, 465 Kajii-cho, Kawaramachi-Hirokoji, Kamigyo-ku, Kyoto 602-8566, Japan; y-hashi@koto.kpu-m.ac.jp (Y.H.); michiaki@koto.kpu-m.ac.jp (M.F.); 4 Department of Diabetes and Endocrinology, Matsushita Memorial Hospital, 5-55, Sotojhima-cho, Morigu-chi-shi 570-8540, Osaka, Japan

**Keywords:** diet, food order, divided carbohydrate, glycemic excursions, continuous glucose monitor, vegetable

## Abstract

**Background/Objectives:** Previous studies show that allocating carbohydrates earlier and vegetables/protein later in late-evening meals improves glycemic control in both healthy individuals and those with type 2 diabetes. However, evidence remains insufficient regarding the effects of distributing carbohydrate intake across the day by dividing three regular meals into five smaller meals. **Methods:** We conducted a randomized, controlled, crossover trial to compare the effects of two dietary patterns: (1) a conventional three-meal pattern with simultaneous intake of all food components, and (2) a five-meal pattern incorporating divided carbohydrate portions and a fixed food order—vegetables first, followed by protein, and then carbohydrates. Eighteen healthy young women consumed the same test meals under both patterns. Glucose fluctuations were monitored using an intermittently continuous glucose monitoring system. **Results:** The five-meal pattern with food sequencing significantly improved the mean amplitude of glycemic excursions (MAGE; 2.56 ± 0.13 vs. 3.49 ± 0.32 mmol/L, *p* < 0.01), glucose peak, and incremental area under the glucose curve for breakfast, lunch, and dinner, and the time above the target glucose range [>7.8 mmol/L; 1.4 ± 0.6 vs. 4.2 ± 1.0%, *p* < 0.01] compared to the three-meal pattern. **Conclusions:** These findings suggest that divided carbohydrate intake and food order ameliorates the MAGE in healthy young women.

## 1. Introduction

Hyperglycemia is a major contributor to increased free radical production and is closely linked to the onset and progression of type 2 diabetes mellitus (T2DM) [1]. Oxidative stress impairs insulin action, creating a vicious cycle in which recurrent postprandial glucose excursions act as key drivers of both microvascular and macrovascular complications. Minimizing postprandial glucose responses may therefore play a critical role in the prevention and attenuation of T2DM [1]. Therefore, medical nutrition therapy is the cornerstone of T2DM management and prevention, encompassing dietary modification, weight control, and physical activity [2,3]. Patients are generally advised to monitor fasting and postprandial glucose levels and adjust their eating patterns to achieve target glycemia, while healthy lifestyle modifications—particularly sustained dietary changes—are also recommended for the long-term prevention of T2DM [4].

A growing body of evidence indicates that both the sequence of food consumption and the fractionation of carbohydrate intake can substantially influence postprandial glucose excursions. Specifically, consuming vegetables first, followed by proteins and carbohydrates last, slows gastric emptying, modulates incretin responses, and blunts rapid postprandial glucose rises among individuals with and without T2DM [5,6,7,8,9]. In both healthy individuals and individuals with T2DM, several reports have demonstrated that late-evening meals elicit smaller glycemic fluctuations when carbohydrate intake is scheduled earlier in the day and vegetables and protein are consumed later [10,11], and that the consumption of snacks or sweets between lunch and dinner, particularly at around 3–4 p.m., can also attenuate postprandial glucose variability [12,13].

A large prospective cohort study demonstrated that diets characterized by a high glycemic index (GI) and high glycemic load (GL) were associated with an increased risk of developing T2DM. In contrast, the findings indicate that adopting dietary patterns with lower GI and GL may help reduce the risk of T2DM onset [14]. Nevertheless, evidence remains limited on whether redistributing an equivalent nutritional load into five smaller meals across the day effectively attenuates postprandial glucose spikes.

To address this gap, we conducted a randomized, controlled, crossover trial to evaluate the effect of a structured meal pattern—five small meals per day, with carbohydrates consumed in divided portions and in a specific food order (vegetables → protein → carbohydrates)—compared with a conventional three mixed meals per day pattern in which all food components were consumed simultaneously. This study, performed in healthy young women, aimed to provide mechanistic insight into how dietary fractionation and food order might attenuate glycemic excursions. We hypothesized that this structured dietary pattern would attenuate postprandial glucose excursions and overall glycemic variability compared with a conventional eating pattern.

## 2. Materials and Methods

### 2.1. Subjects of Experiment

This study, conducted in healthy young Japanese women who were neither pregnant nor diagnosed with type 1 or type 2 diabetes. Female students from Kyoto Women’s University were recruited on a voluntary basis to participate in this study. All participants underwent medical screening, including blood tests for fasting glucose and HbA1c, to confirm the absence of diabetes prior to enrollment. Before participation, they were provided with a comprehensive explanation of the study’s aims, procedures, and any potential risks involved. The exclusion criteria included (1) a clinical diagnosis of type 1 or type 2 diabetes mellitus, (2) the use of medications known to influence glucose metabolism, and (3) current pregnancy or the likelihood thereof. Written informed consent was obtained from each participant prior to study initiation. One week before the intervention began, participants underwent anthropometric measurements and fasting blood sampling to assess baseline plasma glucose and HbA1c levels. Additional demographic and clinical information, including family history of T2DM and use of medications, was also collected. This investigation employed an open-label, randomized, within-subject crossover design. Participants who failed to comply with the study protocol were excluded from the final analysis. The study was conducted in accordance with the ethical standards set forth in the Declaration of Helsinki, received approval from the Clinical Research Ethical Committee of Kyoto Women’s University (Approval Nos. 2023-52), and was registered in the UMIN Clinical Trial Registry (UMIN000057328).

### 2.2. Study Design

**Figure 1:**
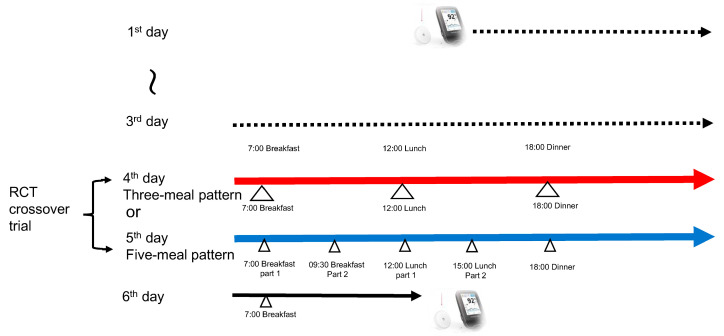
Study protocol. During the study period, each participant wore an intermittent continuous glucose monitoring (isCGM) device for six consecutive days. On the 4th and 5th day, participants consumed identical test meals for three-meal pattern and five-meal pattern in a randomized controlled crossover design. On the sixth day, the isCGM device was removed, and the electronic data collected were compared between the two test days.

This randomized, open-label, within-subject crossover clinical trial was conducted between May and July 2025. Each participant was fitted with an intermittent continuous glucose monitoring (isCGM) device (FreeStyle Libre Pro, Abbott Japan, Tokyo) on the posterior aspect of the left upper arm, under the supervision of the study group, to measure postprandial glucose fluctuations over a 24-h period. Figure 1 and Table 1 present the detailed meal schedule and composition. Standardized test meals were provided, including commercially available dishes—grilled salmon, hamburger steak, and sautéed-vegetable (FamilyMart Co., Ltd., Tokyo, Japan). Additional food items—boiled white rice, white bread, milk, boiled eggs, tomatoes, spinach, broccoli, and simmered turnip greens—were prepared by the participants under the guidance of the research team. The nutritional content of the meals (macronutrients and micronutrients) was the same on both intervention days, differing only in the prescribed eating pattern. Under the randomized crossover protocol, participants consumed the same test meals on the 4th and 5th day, with the order of dietary pattern randomly assigned.

In the conventional eating pattern (three-meal pattern), participants consumed the following:

- Breakfast (07:00–07:15): 120 g of white bread, a boiled egg, broccoli, tomato, strawberry jam (sugar-free), and milk
- Lunch (12:00–12:15): 200 g of boiled white rice, grilled salmon, tomato, boiled spinach, and sautéed-vegetable
- Dinner (18:00–18:15): 200 g of boiled white rice, hamburger steak, boiled spinach, tomato, sautéed-vegetable, and simmered turnip greens
- All meal components were consumed simultaneously within 15 min.

In contrast, the divided eating pattern (five-meal pattern) incorporated a fixed sequence—vegetables first, followed by protein, then carbohydrates—with carbohydrates distributed across two time points:

- Breakfast, Part 1 (07:00–07:15): 60 g of white bread, a boiled egg, broccoli, tomato, strawberry jam (sugar-free), and milk
- Breakfast, Part 2 (09:30–09:45): 60 g of white bread, tomato, broccoli, and strawberry jam (sugar-free)
- Lunch, Part 1 (12:00–12:15): 150 g of boiled white rice, grilled salmon, sautéed-vegetable, tomato, and boiled spinach
- Lunch, Part 2 (15:00–15:15): 100 g of boiled white rice, sautéed-vegetable, and tomato
- Dinner (18:00–18:15): 150 g of boiled white rice, hamburger steak, sautéed-vegetable, tomato, and simmered turnip greens
- All meal components were consumed the food order (vegetables → protein → carbohydrates) within 15 min.

Participants were permitted to drink water, green tea, and unsweetened coffee, but were instructed to avoid alcohol and vigorous physical activity throughout the study period. To ensure compliance, the research team conducted follow-up calls, and participants were instructed to adhere strictly to the provided meal plans and to document all meal-times, dietary intake, and eating behavior. On Day 6, the isCGM devices were removed under supervision at Kyoto Women’s University, and the glucose data were electronically retrieved for analysis. Despite identical meal composition, within-subject comparisons of glycemic responses were conducted for the two eating patterns. The isCGM-derived data were analyzed for additional metrics, including incremental area under the curve (IAUC), glucose peak (GP), standard deviation (SD), maximum glucose level (MAX), minimum blood glucose (MIN), large amplitude of glycemic excursions (LAGE), mean amplitude of glycemic excursions (MAGE), and coefficient of variation (%CV). The IAUC was calculated using the trapezoidal rule, with the starting times set at 7:00 a.m. for breakfast, 12:00 p.m. for lunch, and 6:00 p.m. for dinner [15]. In addition, glycemic metrics including time in range (TIR; 3.9–7.8 mmol/L), time above range (TAR; >7.8 mmol/L), and time below range (TBR; <3.9 mmol/L) were calculated.

### 2.3. Sample Size and Statistical Analysis

The required sample size was determined based on the primary endpoint, the MAGE. We referred to a previous study examining the effect of divided dinner on glycemic excursions in healthy young women [10]. Based on that reference, a minimum of fourteen participants was required to achieve 95% statistical power (G*Power 3.1, Heinrich-Heine-Universität Düsseldorf, Düsseldorf, Germany). Unless otherwise specified, data are presented as means ± standard error of the mean (SEM). The primary outcome was the MAGE, and the secondary outcome was GP. The normality of the data was assessed using the Shapiro–Wilk test, and homogeneity of variances was evaluated using Levene’s test, confirming that the data met the assumptions for parametric analysis. Differences within each group were analyzed using paired *t*-tests, and comparisons between the two groups were performed using independent *t*-tests, with statistical significance set at *p* < 0.05. All statistical analyses were conducted using SPSS Statistics software (version 24; SPSS Japan Inc., Tokyo, Japan).

The macronutritional contents of the test meals for the three-meal pattern and five-meal pattern are shown in Table 1. The participants consumed the same test meals whether consuming three meals or consuming food divided into five meals per day in the RCT crossover study. The total contents and amounts of macronutrients of the test meals were the same on the two days.

## 3. Results

**Figure 2:**
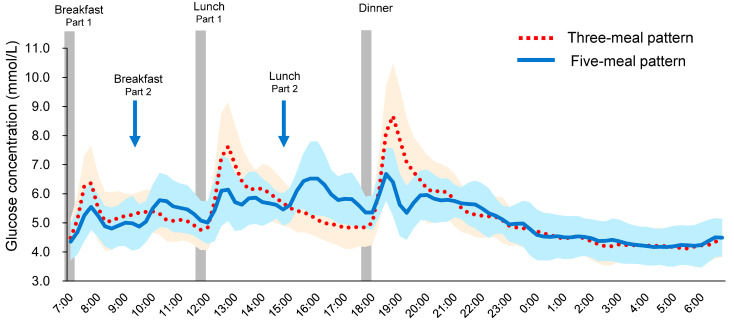
Mean daily blood glucose profiles monitored by isCGM in healthy young women (*n* = 18). Data are presented as mean and the standard deviation (SD). Red dotted line: three-meal pattern; blue solid line: five-meal pattern. Light red area: mean ± SD of three-meal pattern; light blue area: mean ± SD of five-meal pattern.

Eighteen women were enrolled, and all participants completed the trial (Age 21.4 ± 0.8 years, body weight 50.1 ± 5.4 kg, BMI 20.2 ± 1.9 kg/m^2^, HbA1c 5.2 ± 0.2%, fasting blood glucose 4.9 ± 0.3 mmol/L, mean ± SD). Figure 2 shows the mean daily blood glucose profiles measured by isCGM in healthy young women. Postprandial glucose excursions were substantially attenuated with the five-meal pattern compared with the conventional three-meal pattern, which exhibited more pronounced glucose elevations after each meal.

As shown in Table 2, indices of glycemic variability—including SD, MAGE, MAX, LAGE, %CV, GP, and meal-specific IAUC values for breakfast, lunch, and dinner—were significantly lower in the five-meal pattern compared with the conventional three-meal pattern. Time in range above target (TAR) was also significantly lower with the five-meal pattern. In contrast, mean blood glucose levels and the 24-h IAUC did not differ appreciably between the two dietary patterns, likely reflecting the fact that the test meals were matched for total energy, carbohydrate, and other nutrient content.

## 4. Discussion

This study was conducted in healthy young Japanese women who were neither pregnant nor diagnosed with type 1 or type 2 diabetes. The study aimed to examine whether a dietary pattern of five small meals per day—where carbohydrates were consumed in divided portions with carbohydrates consumed last—could attenuate glycemic excursions. We found that this meal pattern effectively reduced postprandial glucose peaks and lowered the MAGE compared with a conventional three-meal pattern. Notably, the time spent above the target glucose range (7.8 mmol/L) was reduced by approximately two-thirds under the five-meal pattern. The reduction in MAGE observed with the five-meal pattern and specific food order strategy in healthy young women suggests a possible primary dietary approach that could help mitigate the onset of T2DM [1,16].

The attenuation of postprandial glucose excursions by the divided carbohydrate pattern can be partially explained by the smaller portions of carbohydrates consumed at each eating occasion [10,11,12,13]. In the five-meal pattern, participants ingested carbohydrates in divided portions—60 g × 2 servings of white bread and 150 g × 2 plus 100 g of boiled white rice—rather than the larger portions of 120 g of white bread and 200 g × 2 of boiled white rice consumed in the three-meal pattern. This effect was particularly pronounced after lunch and dinner, where postprandial glucose levels were markedly lower in the five-meal pattern compared to the three-meal pattern. A prospective cohort study reported that adopting dietary patterns with lower GI and GL may help reduce the risk of T2DM onset [14]. Furthermore, another trial reported that weight loss in both diet groups (low-carbohydrate vs. low-fat) appeared to be driven primarily by a reduction in GL rather than by differences in dietary fat or total caloric intake [17]. Consistent with this, the five-meal pattern in the present study, which involved smaller portions of carbohydrate intake, effectively reduced the overall GL.

Another possible mechanism underlying the amelioration of MAGE is the “second meal phenomenon” [18]. Modest insulin secretion following earlier small meals likely facilitated better glycemic control after subsequent meals. This phenomenon has been attributed to enhanced β-cell responsiveness, where prior glucose exposure primes β-cells for improved insulin secretion during subsequent meals, mediated by β-cell “memory” effects on the first and second phases of insulin release [19,20,21]. Prolonged fasting intervals between meals have been shown to elevate plasma free fatty acid concentrations, which in turn may impair insulin sensitivity. Therefore, dividing meals—particularly carbohydrate intake—into smaller portions, as in the five-meal pattern of this study, reduced the total GL, improved insulin sensitivity, and may have contributed to the attenuation of MAGE.

Moreover, the reduction in postprandial glucose after implementing a five-meal regimen—particularly noticeable at dinner—may relate to circadian regulation of glucose metabolism. Cycles of feeding and fasting modulate transcription of peripheral clock genes as well as genes critical for glucose handling. Previous studies indicate that diet-induced thermogenesis (DIT) can be roughly 40–50% lower at night than in the morning among healthy adults [22]. Disruption of circadian alignment has been linked to insulin resistance, hypertension, and other metabolic disturbances [18,19,20,21,22,23,24,25]. Extending the fasting period before dinner may therefore worsen postprandial hyperglycemia overnight [10,11]. Introducing a modest carbohydrate-split snack in the mid-afternoon (about 15:00–16:00) between lunch and dinner could help stabilize evening glycemia and reduce MAGE [12,13].

The attenuated postprandial glucose excursions observed in this study are likely attributable to the prescribed food order: vegetables first, protein second, and carbohydrates last [4,5,6,7,8]. Dietary fiber from vegetables may form a viscous gel in the stomach, slowing gastric emptying and delaying nutrient delivery to the intestine [5,6,7,8,9,26,27]. This would moderate the rapid rise in blood glucose and incretin responses, resulting in a more gradual absorption of carbohydrates toward the end of the meal. Subsequently, protein intake may stimulate the secretion of incretin hormones, including glucagon-like peptide-1 (GLP-1) and glucose-dependent insulinotropic polypeptide (GIP), which further slow the digestion and absorption of carbohydrates consumed last [11,28,29]. Nevertheless, additional studies are required to clarify these mechanisms in greater detail.

In addition, insulin resistance and β-cell dysfunction are often evident even in the prediabetic state [30]. Hyperglycemia can exacerbate chronic inflammation and promote excessive generation of reactive oxygen species (ROS), leading to vascular dysfunction [30,31]. In turn, oxidative stress and inflammation further aggravate insulin resistance and impair insulin secretion, establishing a self-reinforcing cycle [30,31,32]. The resulting oxidative stress impairs insulin sensitivity, establishing a vicious cycle in which recurrent postprandial glucose surges promote both microvascular and macrovascular dysfunction, even in the pre-diabetic stage [1,16,30,31].

There are several limitations to this study that should be acknowledged. First, the study was conducted in healthy young women to evaluate the effects of consuming meals in a specific food order and in divided portions on glycemic excursions; therefore, the findings may not be generalizable to women with gestational diabetes mellitus (GDM) or other populations. Similar interventional studies are needed in broader cohorts, including individuals with GDM, patients with T2DM, other genders, older adults, and diverse ethnic groups. Second, the study assessed only glycemic parameters and did not evaluate hormonal responses such as insulin, GLP-1, GIP, or other incretin hormones. The underlying mechanisms by which meal sequencing and division attenuate postprandial glucose excursions remain unclear. Large-scale clinical studies are warranted to elucidate these mechanisms and confirm the metabolic effects of structured meal patterns. Third, the study used an isCGM system, which measures glucose concentrations in the interstitial fluid rather than directly in the blood. This may lead to slight discrepancies from plasma glucose levels. Fourth, the test meals reflected typical Japanese dietary patterns, and their macronutrient composition—particularly fat and protein content—may have influenced the rate of glucose appearance and absorption. Thus, the applicability of these findings to populations consuming different types of meals may be limited. Finally, the long-term impact of food order and divided meal consumption on the future risk of T2DM remains uncertain. Future studies should address these questions in diverse populations to confirm the durability and clinical relevance of these findings.

To our knowledge, no prior research has examined whether splitting daily intake into five rather than three eating occasions affects glycemic regulation in healthy young women. In this trial, a regimen of five smaller meals—structured so that carbohydrates were consumed in segmented portions and in a defined sequence (vegetables → protein → carbohydrates)—attenuated postprandial glucose fluctuations compared with the traditional three-meal pattern. This short-term randomized crossover study provides preliminary evidence that dividing meals may help control postprandial hyperglycemia and support metabolic health in healthy individuals, with future studies planned in populations with T2DM and GDM.

## 5. Conclusions

This study is the first to demonstrate that consuming meals in a sequence of vegetables and protein before carbohydrates, combined with dividing carbohydrate throughout the day, significantly ameliorates the MAGE in healthy young women. However, as this study was conducted in young healthy Japanese women, it remains uncertain whether the findings can be generalized to other populations, and caution should be exercised in interpreting the results.

## Figures and Tables

**Table 1:** The contents and amounts of macronutrients of the test meals.

Three-Meal Pattern							Five-Meal Pattern						
	Energy (kcal)	Protein (g)	Fat (g)	Carbohydrate (g)	Fiber (g)	Detail Content		Energy (kcal)	Protein (g)	Fat (g)	Carbohydrate (g)	Fiber (g)	Detail Content
Breakfast	568	28.2	19.2	84.2	9.1	White bread (120 g), tomato, broccoli, a boiled egg, milk, strawberry jam (sugar free)	Breakfast Part 1	385	21.1	16.5	47.0	4.6	White bread (60 g), tomato, broccoli, milk, a boiled egg, strawberry jam (sugar free)
							Breakfast Part 2	183	7.1	2.7	37.2	4.5	White bread (60 g), tomato, broccoli, strawberry jam (sugar free)
Lunch	529	22.0	9.9	93.5	8.6	Boiled white rice (200 g), grilled salmon, sautéed-vegetable, tomato, boiled spinach	Lunch Part 1	426	20.0	9.3	69.7	7.0	Boiled white rice (150 g), grilled salmon, sautéed-vegetable, tomato, boiled spinach
							Lunch Part 2	205	4.0	1.0	47.3	3.3	Boiled white rice (100 g), sautéed-vegetable, tomato
Dinner	725	27.6	23.6	106.8	9.5	Boiled white rice (200 g), hamburger steak, sautéed-vegetable, tomato, simmered turnip greens	Dinner	623	25.6	23.1	83.3	7.8	Boiled white rice (150 g), hamburger steak, sautéed-vegetable, tomato, simmered turnip greens
Total	1822	77.8	52.6	284.5	27.2		Total	1822	77.8	52.6	284.5	27.2	

**Table 2:** Glycemic parameters of three-meal pattern and five-meal pattern in healthy young women (*n* = 18).

	Three-Meal Pattern	Five-Meal Pattern
MBG (mmol/L)	5.25 ± 0.14	5.20 ± 0.14
SD (mmol/L)	1.07 ± 0.08	0.84 ± 0.04 **^†^
MAGE (mmol/L)	3.49 ± 0.32	2.56 ± 0.13 **^†^
MAX (mmol/L)	8.96 ± 0.40	7.35 ± 0.25 ***^††^
MIN (mmol/L)	3.85 ± 0.15	3.92 ± 0.17
LAGE (mmol/L)	5.11 ± 0.41	3.43 ± 0.18 ***^††^
%CV (%)	1.14 ± 0.09	0.91 ± 0.06 ***^†^
Breakfast GP (mmol/L)	6.60 ± 0.27	5.88 ± 0.19 **^†^
Lunch GP (mmol/L)	7.92 ± 0.32	6.68 ± 0.21 ***^††^
Dinner GP (mmol/L)	8.93 ± 0.40	7.04 ± 0.20 ***^†††^
Breakfast		
IAUC 60 min (mmol/L × min)	72 ± 8	44 ± 6 **^††^
IAUC 120 min (mmol/L × min)	115 ± 13	80 ± 9 **^†^
IAUC 180 min (mmol/L × min)	167 ± 17	125 ± 13 *
IAUC 240 min (mmol/L × min)	211 ± 18	200 ± 16
IAUC 300 min (mmol/L × min)	241 ± 21	257 ± 21
Lunch		
IAUC 60 min (mmol/L × min)	108 ± 13	46 ± 7 ***^†††^
IAUC 120 min (mmol/L × min)	198 ± 24	93 ± 14 ***^††^
IAUC 180 min (mmol/L × min)	259 ± 25	134 ± 18 ***^†††^
IAUC 240 min (mmol/L × min)	290 ± 25	212 ± 28 **
IAUC 300 min (mmol/L × min)	303 ± 27	275 ± 36
Dinner		
IAUC 60 min (mmol/L × min)	141 ± 14	50 ± 7 ***^†††^
IAUC 120 min (mmol/L × min)	250 ± 29	79 ± 12 ***^†††^
IAUC 180 min (mmol/L × min)	314 ± 35	114 ± 19 ***^†††^
IAUC 240 min (mmol/L × min)	344 ± 36	138 ± 20 ***^†††^
IAUC 300 min (mmol/L × min)	361 ± 37	148 ± 24 ***^†††^
24 h IAUC (mmol/L × min)	1236 ± 97	1319 ± 99
TIR (%) 3.9–7.8 mmol/L	86.9 ± 4.6	90.3 ± 3.6
TBR (%) < 3.9 mmol/L	8.9 ± 4.7	8.3 ± 3.7
TAR (%) > 7.8 mmol/L	4.2 ± 1.0	1.4 ± 0.6 **^†^

The data are the mean ± SEM. MBG, mean blood glucose; SD, standard deviation for glucose; MAGE, mean amplitude of glycemic excursion; MAX, maximum blood glucose; MIN, minimum blood glucose; LAGE, large amplitude of glycemic excursion; %CV, coefficient of variation; GP, glucose peak; IAUC, incremental area under the curve for glucose. Time in range (TIR; 3.9–7.8 mmol/L), time below range (TBR; <3.9 mmol/L), and time above range (TAR; >7.8 mmol/L). Three-meal pattern vs. five-meal pattern; *p* < 0.05 *, *p* < 0.01 **, *p* < 0.001 *** according to paired *t*-test. Three-meal pattern vs. five-meal pattern; *p* < 0.05 ^†^, *p* < 0.01 ^††^, *p* < 0.001 ^†††^ according to *t*-test.

## Data Availability

The original contributions presented in this study are included in the article. Further inquiries can be directed to the corresponding author.
